# On the Treatment of Obstruction of the Bowels

**Published:** 1851-12

**Authors:** 


*On the Treatment of Obstruction of the Bowels.* By Edward Wells, M. D., Oxon.

[In some preliminary remarks, the author informs us that it is not his object to treat of intestinal obstructions from causes external to the tube, as tumors, &c., nor of obstructions arising from internal causes, as hardened fæces, neither of those cases which originate in hernia. The cases which he has in view are those which have no demonstrable cause of the obstruction, such as in the following supposed case.]

You are called to a patient, who informs you that he has had no proper relief from the bowels for the last seven or eight days; that he has been to the druggist, and taken black dose upon black dose, pill upon pill, and that they are all in him, and he wants to know what he is to do next. He tells you further, that it is true he has been to stool once or twice, or, perhaps even oftener, during the time, that he has, on each occasion, passed something, but he is sure it is not what he ought to have passed. In short, to use his own expression, although he has occasionally had a scanty evacuation, he is convinced that "*nothing has gone through him.*" Upon examining the abdomen, you find some distension around the umbilicus, with a degree of tenderness on pressure. This last symptom varies from that slight shade on which the patient can hardly say whether the pressure relieves his pain or not, up to decided tenderness on the least touch. In mild cases, the patient will tell you he feels very well, excepting the obstruction, but the knowledge of its existence makes him very uncomfortable. In other cases there is some degree of sickness conjoined, merely, perhaps, occasioned by the purgative draughts. In severer cases the sickness is more permanent, mucus or bile being rejected from the stomach. In such instances we should expect the tenderness on pressure over the bowels, to be greater, though still not in any degree approaching to what usually occurs in peritonitis. All this time the pulse is not perhaps accelerated, it is generally weak; the tongue is moist and often clean; the urine, provided the obstruction be not situated high up in the bowels, is not necessarily affected, though generally high colored.

Under these circumstances, and especially in the milder cases, the first thing, perhaps, that you do, is to order a large enema to be thrown up. It is found to traverse the large intestine easily; the patient assures you that he feels it go as far as the ileo-cœcal valve, and after a short time it returns without any tinge of fæcal matter. The obstruction is not in any part of the colon, but somewhere in the small intestine.

What treatment should then be adopted? In the severer cases, where there is pain upon pressure, distension of a portion of the intestine, a rumbling of flatus, and frequent vomiting, it will be said that the line of treatment is easily chalked out; that, whatever the cause of the obstruction, we have inflammation superadded; and that our treatment must be directed to subdue the latter. This is quite true: and, in such well-marked cases, I do not think there would be much chance of the case being misunderstood. But we must remember that these severe instances of the disease are only the consequence of a continuation and aggravation of the symptoms of its milder forms. We must not forget that the most simple case of obstruction is liable to run on into a fatal form, if, with the view of obtaining an action of the bowels, we are incautious in the prolonged use of irritating medicines. Finding that the patient's chief discomfort arises from the fact of the bowels not acting, that he professes himself as feeling otherwise well, we are, perhaps, rather too liable to fall in with his own fancies, and just give him one more dose.

Now, in these cases, what ought we to do? In the first place, abstain entirely from all purgative medicine. It will be much better to err in not giving sufficient aperients, than to err in giving too much. The first thing to do is to compose the patient's mind by informing him that there is no hurry for the bowels to act; that, if he waits patiently, they will be sure to act in time; to tell him instances of persons who have gone a long time without any action of the bowels, and have done well.

Next, in these cases of obstinate obstruction, I have great faith in the lancet, where it can with safety be used. It has seemed that a slight degree o faintness produced by bloodletting, has acted very beneficially in removing the exciting causes of the obstruction, probably by the general relaxation which the faintness itself occasions. By putting the patient in an upright position, and bleeding him until he begins to feel slightly faint, I think we are quite safe not to do him any harm. If he is of a weak, nervous temperament, a very few ounces will produce the desired effect. If he be strong, he will afford to lose more. Where, however, the debility of the patient forbids the use of the lancet, it will be as well to apply leeches around the umbilicus. These act, probably, by relieving the local congestion, which is either the cause or the effect of the obstruction.

These measures premised, the safest plan is, I think, to put the patient upon repeated doses of calomel and opium. Even if inflammation be totally absent, the exhibition of these two drugs is likely to be attended with the best effects. The opium soothes the bowels already irritated by the repeated cathartics: it allays the over-excited peristaltic action; it relaxes any contingent spasm, and quiets the patient's mind. To effect these objects, it must be administered in sufficient doses — such as gr. ½ to gr. j. every four hours. The * calomel, by improving the secretions, and exciting the action of the liver, tends to remove the cause of the obstruction. And if this happen to depend upon a partial enteritis, the combined action of these two medicines would hold out the best hopes of a successful treatment. If the calomel be sufficiently guarded by opium, there is not, I think, any fear of its producing any serious irritation of the bowels.

While using these remedies, I should be in no hurry to accelerate the action of the bowels by aperients. I should rather wait until they begin to act of themselves, as they generally will; and then, provided no inflammatory symptoms were present, there would be no objection to administer a dose of castor-oil to aid their propulsive efforts. In these cases it is also better to delay the administration of aperient enemata until the bowels are acting of themselves. Previously to this, they appear to add rather to the patient's discomfort, probably by the distension they occasion in the large intestine, which reacts upon the parts already distended by the obstruction.

When there is no tendency to sickness, it is better to allow the patient to take food, in the shape of gruel, by the mouth. It prevents that sense of sinking which he often experiences, and it probably acts in some degree mechanically in propelling the contents of the intestinal tube.

In those severer cases, where there is frequent sickness, with pain in the bowels, and a rumbling of flatus, the above measures will be still further indicated. But there will be also other things which it will then be necessary to attend to. In these cases it is of great importance to abstain from giving any food by the mouth for some days. A teaspoonful of cold water should be put into the mouth from time to time to allay the patient's thirst. His support should be entirely entrusted to beef-tea injections. It is proved that these are sufficient to maintain the strength for some time — at any rate, for a period sufficient to allay the irritating symptoms, which forbid the exhibition of food by the mouth. This part of the treatment I am inclined to consider as of the highest importance; for, as long as food is continued to be

* The calomel might as well be omitted. To "improve secretions" and "excite the liver," are expressions which it is to be hoped will soon become obsolete.—*Editor Buffalo Medical Journal.*

administered by the mouth, and is rejected by vomiting, there will be little chance of arresting the inversion of the peristaltic action of the intestinal tube. The nutritive enemata should be of small bulk, not exceeding, at the outside, a quarter of a pint; otherwise they will not only be retained, but they will add to the patient's sufferings. They should be administered at regular intervals of four hours. When there is much rumbling of the intestines, or when there is a difficulty as to the retention of the injections, it is advisable to add to them a certain proportion of laudanum.— *Medical Gazette, Ranking's Abstract.*

